# Cinacalcet inhibits neuroblastoma tumor growth and upregulates cancer-testis antigens

**DOI:** 10.18632/oncotarget.7448

**Published:** 2016-02-17

**Authors:** Carlos J. Rodríguez-Hernández, Silvia Mateo-Lozano, Marta García, Carla Casalà, Ferran Briansó, Nerea Castrejón, Eva Rodríguez, Mariona Suñol, Angel M. Carcaboso, Cinzia Lavarino, Jaume Mora, Carmen de Torres

**Affiliations:** ^1^ Developmental Tumor Biology Laboratory, Institut de Recerca Pediàtrica - Hospital Sant Joan de Déu, Esplugues de Llobregat, Barcelona, Spain; ^2^ Statistics and Bioinformatics Unit, Vall d'Hebron Research Institute, Barcelona, Spain; ^3^ Department of Pathology, Institut de Recerca Pediàtrica - Hospital Sant Joan de Déu, Esplugues de Llobregat, Barcelona, Spain; ^4^ Department of Oncology, Institut de Recerca Pediàtrica - Hospital Sant Joan de Déu, Esplugues de Llobregat, Barcelona, Spain

**Keywords:** neuroblastoma, calcium-sensing receptor, cinacalcet, ER stress, cancer-testis antigens

## Abstract

The calcium–sensing receptor is a G protein-coupled receptor that exerts cell-type specific functions in numerous tissues and some cancers. We have previously reported that this receptor exhibits tumor suppressor properties in neuroblastoma. We have now assessed cinacalcet, an allosteric activator of the CaSR approved for clinical use, as targeted therapy for this developmental tumor using neuroblastoma cell lines and patient-derived xenografts (PDX) with different *MYCN* and *TP53* status. *In vitro*, acute exposure to cinacalcet induced endoplasmic reticulum stress coupled to apoptosis via ATF4-CHOP-TRB3 in CaSR-positive, *MYCN*-amplified cells. Both phenotypes were partially abrogated by phospholipase C inhibitor U73122. Prolonged *in vitro* treatment also promoted dose- and time-dependent apoptosis in CaSR-positive, *MYCN*-amplified cells and, irrespective of *MYCN* status, differentiation in surviving cells. Cinacalcet significantly inhibited tumor growth in *MYCN*-amplified xenografts and reduced that of *MYCN*-non amplified PDX. Morphology assessment showed fibrosis in *MYCN*-amplified xenografts exposed to the drug. Microarrays analyses revealed up-regulation of cancer-testis antigens (CTAs) in cinacalcet-treated *MYCN*-amplified tumors. These were predominantly CTAs encoded by genes mapping on chromosome X, which are the most immunogenic. Other modulated genes upon prolonged exposure to cinacalcet were involved in differentiation, cell cycle exit, microenvironment remodeling and calcium signaling pathways. CTAs were up-regulated in PDX and *in vitro* models as well. Moreover, progressive increase of CaSR expression upon cinacalcet treatment was seen both *in vitro* and *in vivo*. In summary, cinacalcet reduces neuroblastoma tumor growth and up-regulates CTAs. This effect represents a therapeutic opportunity and provides surrogate circulating markers of neuroblastoma response to this treatment.

## INTRODUCTION

Neuroblastic tumors encompass a heterogeneous group of developmental malignancies of the sympathetic nervous system that include the neuroblastomas, ganglioneuroblastomas and ganglioneuromas [1]. Disseminated neuroblastomas in infants and local-regional, well-differentiated tumors are usually benign. However, high-risk tumors, i.e. metastatic neuroblastomas in patients older than 18 months and some local-regional cases display a notable capacity to recur and/or become refractory and, unfortunately, there is no therapy known to be curative for them [2].

Several genetic and epigenetic abnormalities have been associated with this variety of clinical presentations, including alterations of ploidy, chromosomal aberrations, *MYCN* amplification and mutations of *ALK* [3-5]. However, few of them are actionable [5]. Long before many of them were described, differentiated neuroblastic tumors were reported to be associated with favorable outcome [6]. Cytodifferentiation can be pharmacologically induced in neuroblastoma models [7-10] and, owing to its differentiating properties, retinoic acid has become part of the standard of care of high-risk neuroblastomas [11].

Our group described a gene potentially involved in the differentiation pathways of neuroblastic tumors, the calcium-sensing receptor (CaSR). CaSR was first reported as a family C G-protein coupled receptor (GPCR) that senses plasmatic fluctuations of Ca^2+^ and regulates the secretion of parathyroid hormone accordingly [12]. This GPCR is also expressed in many organs not involved in calcium homeostasis and in some neoplasias in which it plays cell-type specific functions [13]. Our initial work showed that CaSR is expressed in benign, differentiated neuroblastic tumors and up-regulated upon differentiation induction [14]. Next, we reported that the *CaSR* gene is silenced by genetic and epigenetic mechanisms in *MYCN*-amplified, unfavorable neuroblastomas [15]. Accordingly, ectopic overexpression of the CaSR significantly reduced the proliferative and tumorigenic capacities of neuroblastoma cells. Moreover, CaSR-positive cells underwent apoptosis upon acute reactivation of the receptor with calcium, its main orthosteric ligand [15]. In keeping with these findings, a tri-locus haplotype containing a moderately inactivating variant of the CaSR was associated with undifferentiated histology, metastatic disease and poor outcome [16].

Altogether, our published data were consistent with the hypothesis that the CaSR exerts tumor-suppressor functions in neuroblastoma. Thus, we next sought to evaluate whether cinacalcet, an allosteric activator of the CaSR approved for clinical use [17], might reduce neuroblastoma tumor cell growth. Based on our previous work, we hypothesized that it could promote cytodifferentiation and/or cell death.

## RESULTS

### Acute exposure to cinacalcet triggers apoptosis in neuroblastoma cells

Among available neuroblastoma cell lines, only LA-N-1 cells exhibited detectable endogenous *CaSR* mRNA expression [15]. Low levels of CaSR protein were present in these cells as well (Supplementary Figure S1A). Two *MYCN*-amplified cell lines were previously transfected with pCMV-CaSR-GFP or pCMV-GFP [15]. CaSR protein expression was also present in the neuroblastoma metastasis from which a PDX model was generated (Supplementary Figure S1A). However, this model did not grow *in vitro* and therefore was only used for *in vivo* studies. A second *MYCN*-non amplified model was generated for *in vitro* analyses by stable transfection of SH-SY5Y cells (Supplementary Figure S1B and S1C).

**Figure 1:**
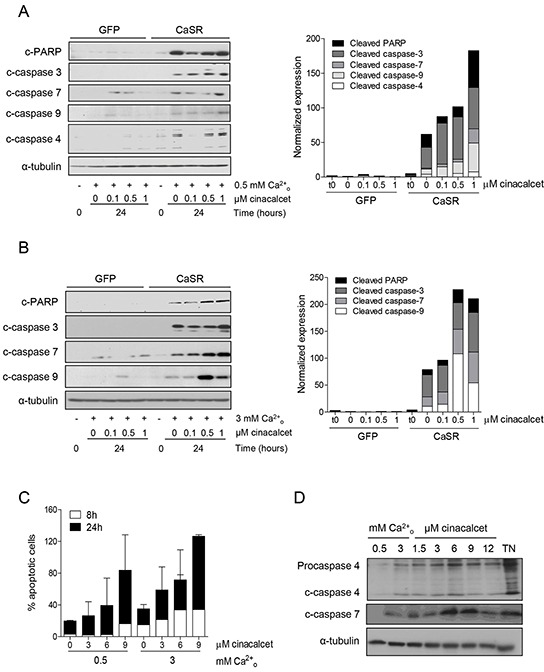
Acute exposure to cinacalcet induces apoptosis in neuroblastoma cells **A.** SK-N-LP cells stably transfected with pCMV-GFP or pCMV-CaSR-GFP were grown in serum deprivation media for 16 hours. They were then exposed to cinacalcet for 24 hours at indicated doses in the same media containing 0.5 mM CaCl_2_. Total proteins were isolated from floating and adherent cells to conduct immunoblots. **B.** The same experiment was performed in the presence of 3 mM CaCl_2_. **C.** LA-N-1 cells were exposed to cinacalcet for 8 or 24 hours following serum deprivation, in the presence of 0.5 or 3 mM CaCl_2_. Cells were collected and stained with Annexin V-FITC and propidium iodide. Apoptosis was quantified by flow cytometry. Data presented are an average of two independent experiments. Error bars represent standard error of mean (SEM). Increase of apoptotic cells upon cinacalcet exposure was not statistically significant (two-tailed Student's *t*-test). **D.** LA-N-1 cells were exposed to cinacalcet for 8 hours as in panel C. Cells were collected and immunoblots were performed. As a positive control of ER stress induced caspase-4 activation, LA-N-1 cells were exposed to 5 μg/mL tunicamycin (TN). Panels A, B and D show representative results of at least three independent experiments.

We have previously reported that acute exposure to high extracellular calcium (Ca^2+^_o_) concentrations following serum deprivation [18] induces apoptotic cell death in CaSR-overexpressing neuroblastoma cells [15]. To assess whether cinacalcet is able to increase this effect, SK-N-LP cells were exposed to DMSO or cinacalcet in serum deprivation media. As shown in Figure 1, cinacalcet (doses ranging between 0.1 and 1 μM) increased cleavage of PARP and caspases-4, -3, -7 and -9 produced by either 0.5 (Figure 1A) or 3 mM CaCl_2_ (Figure 1B) in CaSR-overexpressing cells in a time- (not shown) and dose-dependent manner, even if 3 mM CaCl_2_ was already a potent apoptotic stimulus.

In LA-N-1 cells, cinacalcet also induced apoptosis in a time- and dose-dependent manner. Analyses by flow cytometry of annexin V-propidium iodide stained cells showed a not statistically significant increase of apoptotic cells upon exposure to cinacalcet (Figure 1C). This effect was only seen at high doses, in accordance with the reduced levels of CaSR expression present in this cell line.

These doses of cinacalcet also prompted increased cleavage of caspases-4 and -7 in LA-N-1 cells, an additional evidence of ER stress mediated apoptosis (Figure 1D). In these experiments, tunicamycin (TN) was used as a positive control of ER-stress induced activation of caspase-4, an effect promoted by its capacity to induce Ca^2+^ exit from the ER [19].

### Cinacalcet-induced apoptosis in *MYCN*-amplified neuroblastoma cells is prompted by ER stress and both are dependent on activation of phospholipase C

**Figure 2:**
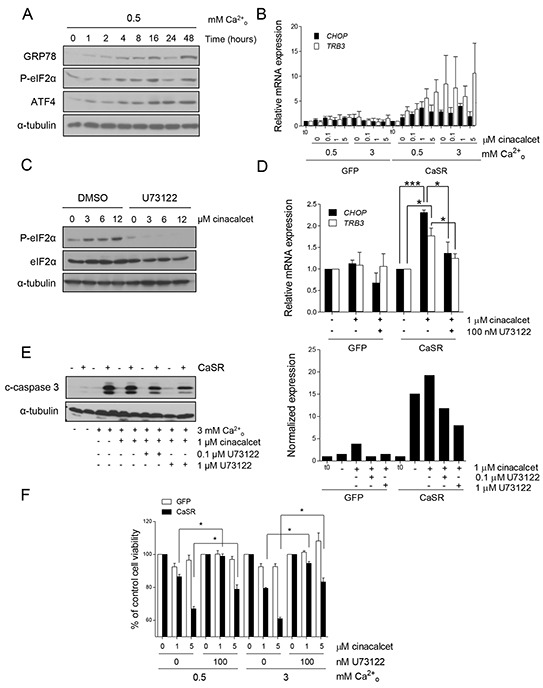
Apoptosis upon cinacalcet exposure is triggered by phospholipase C activation and ER stress **A.** SK-N-LP cells stably transfected with pCMV-CaSR-GFP were exposed to 6 μM cinacalcet following serum deprivation in 0.5 mM CaCl_2_ for indicated periods of time and lysed for Western blot analyses. **B.** CaSR-positive and –negative SK-N-LP cells were exposed to indicated concentrations of cinacalcet for 24 hours following serum deprivation, as described in A. Total RNA was isolated to analyze *CHOP* and *TRB3* mRNA expression by RT-qPCR. Data are presented as an average of two independent experiments. Error bars represents SEM. Increase of transcripts levels is not statistically significant, two-tailed Student's *t*-test. **C.** LA-N-1 cells were treated with cinacalcet at indicated doses for 2 hours, in the presence or absence of 10 μM U73122 or DMSO, and lysed for Western blot analysis. **D.** SK-N-LP cells stably transfected with pCMV-GFP or pCMV-CaSR-GFP were exposed to cinacalcet for 24 hours following serum deprivation, in the presence or absence of 100 nM U73122. Relative mRNA expression levels of *CHOP* and *TRB3* were analyzed by RT-qPCR. Data are presented as an average of three independent experiments. Error bars represents SEM. *P < 0.05, *** *P* < 0.0001, two-tailed Student's *t*-test. **E.** CaSR-positive and -negative SK-N-LP cells were exposed to 3 mM CaCl_2_ and 1 μM cinacalcet in the presence or absence of 0.1 or 1 μM U73122 for 24 hours before collecting cells for immunoblot analyses. Bands intensity was quantified relative to that of α-tubulin (right). **F.** SK-N-LP cells stably transfected with pCMV-GFP or pCMV-CaSR-GFP were exposed to indicated doses of cinacalcet in either 0.5 or 3 mM CaCl_2_ following serum deprivation, in the presence or absence of 100 nM U73122. Cell viability was measured 48 hours later by MTS assays. Data are presented as an average of two independent experiments. Error bars represents SEM. *P < 0.01, two-tailed Student's *t*-test. Panels A, C and E show representative results of three independent experiments.

We hypothesized that apoptosis following acute exposure to cinacalcet might be prompted by activation of phospholipase C (PLC), IP_3_-mediated calcium leak from the endoplasmic reticulum (ER), and consequent ER stress. Consistent with this mechanism, increased levels of GRP78, ATF4 and P-eIF2α (Figure 2A), as well as a not statistically significant up-regulation of *CHOP* and *TRB3* mRNA (Figure 2B), were detected in neuroblastoma cells following cinacalcet exposure in a time-, dose-, calcium- and CaSR-dependent manner (Supplementary Figure S2A and S2B).

Moreover, PLC inhibitor U73122 abrogated phosphorylation of eIF2α in LA-N-1 cells at all doses of cinacalcet examined (Figure 2C). U73122 also significantly attenuated up-regulation of *CHOP* and *TRB3* in CaSR-overexpressing cells exposed to cinacalcet (Figure 2D). More importantly, cleavage of caspase-3 (Figure 2E) and decreased cell viability (Figure 2F) induced by cinacalcet in CaSR-positive neuroblastoma cells were significantly reduced by U73122.

As expected [20], ER stress and consequent apoptosis were not induced in *MYCN*-non amplified SH-SY5Y cells (Supplementary Figure S2C and S2D).

### Chronic exposure to cinacalcet induces apoptosis and differentiation in surviving neuroblastoma cells

A concentration-dependent decrease of cell viability was observed in neuroblastoma cell lines exposed to cinacalcet or another calcimimetic, NPS R-568. Half maximal inhibitory concentration (IC_50_) values of cinacalcet were significantly lower than those of NPS R-568 in most cases. However, similar IC_50_ values were obtained in human fibroblasts and HEK-293 cells (Supplementary Figure S3A).

When the same neuroblastoma cell lines were allowed to proliferate in the presence of concentrations lower than IC_50_ for 5 days, a moderate dose- and time-dependent decrease of cell viability was detected in CaSR-positive cells (Supplementary Figure S3B). This was only seen in CaSR-negative cells at higher concentrations (Supplementary Figure S3C). This phenotype was rescued by pan-caspase inhibitor Z-VAD-FMK (Supplementary Figure S3D and S3E), but differences between CaSR-positive and –negative cells were modest.

**Figure 3:**
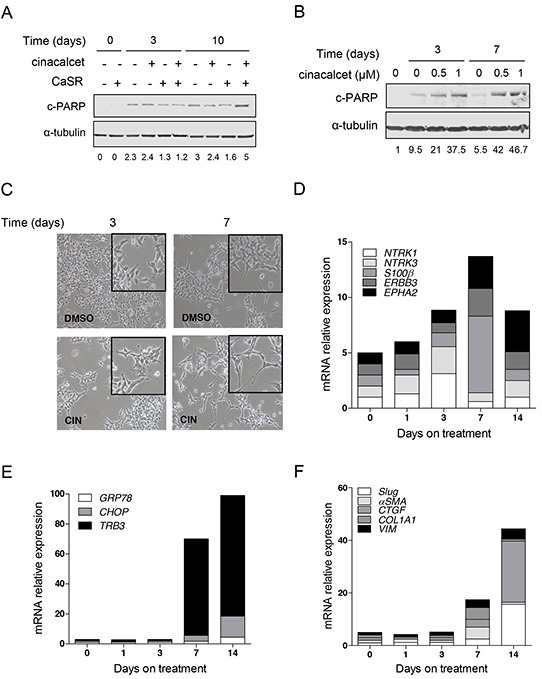
Prolonged *in vitro* exposure to cinacalcet induces apoptosis and cytodifferentiation in surviving neuroblastoma cells **A.** CaSR-positive and -negative SK-N-LP cells were grown in the presence of 1 μM cinacalcet or DMSO. Cells were lysed at indicated times to perform immunoblots. Bands intensity was quantified relative to that of α-tubulin. Blots shown are representative of three independent experiments. **B.** LA-N-1 cells were grown in the presence of indicated doses of cinacalcet or DMSO. Cells were lysed at indicated times to perform immunoblots. Data shown are representative of three independent experiments. **C.** Microphotography of LA-N-1 cells grown in the presence of 1 μM cinacalcet or DMSO for 3 and 7 days. **D.** Total RNA was isolated from LA-N-1 cells exposed to 1 μM cinacalcet or DMSO at indicated days. Gene expression analyses were carried out by RT-qPCR. Transcript levels were quantified relative to those detected in cells exposed to DMSO for the same number of days, and compared to time 0 (cells collected 16 hours after plating). Graph shows markers involved in neuroblastoma differentiation. **E.** Graph showing relative expression levels of transcripts involved in ER stress in cells processed as in panel D. **F.** Plot of transcripts involved in EMT analyzed as in panel D. See also Table 1 to compare gene expression patterns in three cell lines exposed to 1 μM cinacalcet or DMSO for 14 days. Data showed in panels C, D, E and F are representative of at least two independent experiments for each cell line.

Next, LA-N-1 cells and SK-N-LP and SH-SY5Y transfected models were cultured in the presence of 0.5-1 μM cinacalcet or DMSO for 14 days. Increased cleavage of PARP was seen in cinacalcet-treated cells and this output was dependent on CaSR expression, time of exposure (Figure 3A) and dose (Figure 3B). In surviving cells, morphological features of neuronal differentiation such as extensive neurite outgrowth were already apparent at day 3 and reached maximal expression between days 7 and 14 (Figure 3C). Several transcripts associated with cytodifferentiation (Figure 3D), cell cycle exit, ER stress (Figure 3E) and epithelial-to-mesenchymal transition (EMT) (Figure 3F) were concomitantly up-regulated, as evaluated by RT-qPCR (Table 1). Increased expression of transcripts associated with ER stress was mostly seen in *MYCN*-amplified cells (Table 1). Also, higher induction of differentiation markers was detected in SK-N-LP cells, which exhibit a less differentiated phenotype than LA-N-1 and SH-SY5Y cell lines (Table 1) [21]. In keeping with differentiation induction, *MYCN* mRNA levels decreased in *MYCN*-amplified cells, together with down-regulation of inhibitor of differentiation 2 (*ID2*) and reverse transcription telomerase (*TERT*) in the three models. Interestingly, up-regulation of *CaSR* mRNA was seen in the three cell lines exposed to cinacalcet (Table 1).

**Table 1:** Gene expression analyses of neuroblastoma cell lines following *in vitro* exposure to cinacalcet conducted by RT-qPCR

Phenotype	Gene	*MYCN*-A		*MYCN*-NA
		LA-N-1	SK-N-LP	SH-SY5Y
	*CaSR*	2.5	**45.7**	4
Proliferation	*MYCN*	3.3	8.9	2.4
	*ID2*	2.9	1.6	3
	*TERT*	6.8	9.1	1.5
Differentiation	*NFL*	1.8	7.2	ns
	*TUBB3*	2.5	2.8	ns
	*S100-β*	6.9	**72.3**	1.7
	*EPHA2*	3.7	1.8	**12.1**
	*NTRK1*	3.1	3.6	1.3
	*NTRK3*	2.4	2.6	1.4
	*p75/NTR*	1.4	3.5	3.6
ER stress, apoptosis	*GRP78*	4.4	6.4	1.8
	*CHOP*	**14.1**	**21.9**	5.2
	*TRB3*	**71.8**	**19.6**	2.6
	*BAX*	1.8	6.7	1.5
EMT	*Snail1*	2	10.9	7.5
	*Slug*	**15.6**	1.6	**30.1**
	*TGF-β*	2.1	2.7	1.4
	*TGF-β2*	4.4	**11.4**	1.5
	*α-SMA*	4.5	**41.1**	4.9
	*CTGF*	**23.2**	3.4	35.4
	*COL1A1*	4.5	1.8	2.1
	*COL3*	**20.8**	2.5	**31.7**
	*VIM*	3.9	5.3	2.3
	*VCAN*	**14.2**	6.6	2.9
	*FN*	1.9	1.6	2.8
CTAs	*CTCFL*	no expression	no expression	no expression
	*SSX4/4B*	3.8	**69**	5.3
	*GAGE12-P1*	2.7	4.1	3.2
	*GAGE12-P2*	7.6	5.7	5.6
	*GAGE12-P3*	5.1	6.3	1.6
	*MAGE-A2*	2.9	**36.6**	1.4
	*MAGE-A3*	1.4	**155.4**	1.5
	*NY-ESO-1*	4.2	**10.8**	3.1
Calcium signaling	*PRKCA*	3	2.7	9.1
	*RYR2*	4.7	10	3.3
	*ADCY8*	7	**81.9**	3.7
**Fold change**	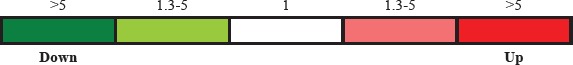				

Bold: Fold change > 10

*MYCN*-A: *MYCN*-amplified; *MYCN*-NA: *MYCN*-non amplified.

EMT: Epithelial-to-mesenchymal transition.

CTAs: Cancer-testis antigens.

### Cinacalcet inhibits neuroblastoma tumor growth *in vivo* and upregulates cancer-testis antigens

**Figure 4:**
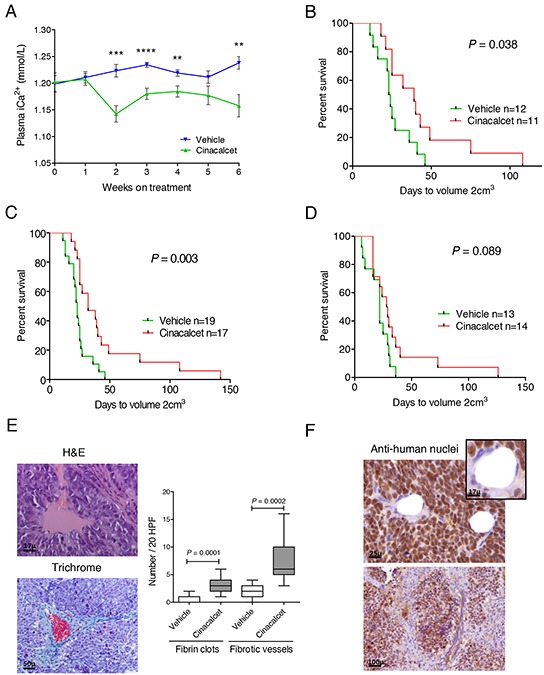
Cinacalcet inhibits neuroblastoma tumor growth LA-N-1 cells (10^7^) or patient-derived xenografts fragments were subcutaneously inoculated in four to six-week-old female athymic Nude-Foxn1 *nu*/*nu* mice. Tumors were allowed to grow until dimensions reached 7×7 mm. Mice were then randomized to receive either vehicle or cinacalcet (10 mg/kg/day) by oral gavage 6 days per week until tumor volume reached 2 cm^3^. **A.** Blood samples (100 μL) were collected from facial veins and plasma ionic Ca^2+^ concentrations (mmol/L) measured once a week ** *P* < 0.01; *** *P* < 0.001; **** *P* < 0.0001, two-tailed Student's *t*-test. **B.** Event-free survival (EFS) rates of mice bearing LA-N-1 xenografts that received either cinacalcet or vehicle. The log-rank statistic was used to compare EFS probabilities between groups. **C.** Pooled data of two independent experiments performed as in B. **D.** Immunocompromised mice bearing *MYCN*-non amplified, patient-derived xenografts received vehicle or cinacalcet as above. EFS probabilities were compared as in panel B. **E.** Haematoxylin-eosin (left, upper panel) and Masson's trichrome (left, lower panel) of formalin fixed, paraffin-embedded sections of LA-N-1 tumors treated with cinacalcet. Right: Number of fibrin clots and fibrotic vessels in 20 high power fields were counted on H&E (clots) or trichrome (fibrotic vessels) stained sections of LA-N-1 xenografts exposed to cinacalcet or vehicle as in panel C. Means were compared by two-tailed Student's *t*-test. **F.** Immunohistochemistry performed with anti-human nuclei antibody in sections of LA-N-1 xenografts exposed to cinacalcet.

Immunocompromised mice carrying xenografts with different *MYCN* and *TP53* status received either vehicle or cinacalcet until tumors reached 2 cm^3^. Mild, non-symptomatic, hypocalcemia was documented (Figure 4A). No signs of toxicity were observed (not shown).

In the first experiment conducted with *MYCN-*amplified, *TP53*-null xenografts, significant tumor growth inhibition was observed (Figure 4B). This experiment was replicated and pooled data showed higher statistical significance (Figure 4C). Inhibition of PDX growth showed a similar tendency (Figure 4D).

Fibrin clots and heavy collagen deposition around tumor vessels were identified in LA-N-1 xenografts following cinacalcet treatment (Figure 4E). Both findings were significantly less frequent in cinacalcet-treated PDX (not shown). Occasionally, fibrosis invaded vast areas of LA-N-1 specimens, but no evidence of fibrosis was found in normal organs (not shown). Immunohistochemistry revealed that most cells involved in microenvironment remodeling, including tumor vessels and fibroblasts, were of murine origin (Figure 4F). Thus, microarrays analyses and RT-qPCR were conducted to analyze human and murine transcripts.

**Figure 5:**
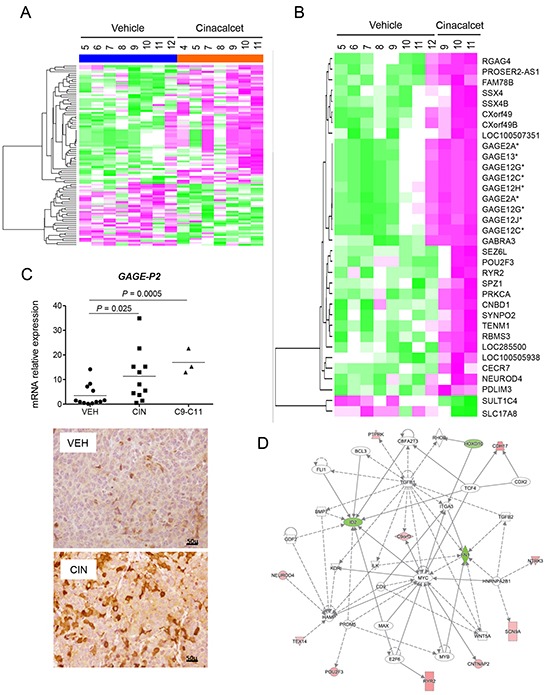
Genome-wide expression analyses of cinacalcet- and vehicle-treated neuroblastoma xenografts reveals up-regulation of cancer-testis antigens **A.** Heat map comparing gene expression patterns in LA-N-1 xenografts exposed to vehicle or cinacalcet in the first survival experiment. These were the last tumors excised in each group. **B.** Heat map comparing gene expression patterns in the 8 control LA-N-1 xenografts and the three xenografts exposed to the longest treatment with cinacalcet. **C.** Upper panel: Relative expression of *GAGE* mRNA in control and cinacalcet-treated LA-N-1 tumors. Statistical significance was calculated using two-tailed Mann-Whitney *U* test to compare all cinacalcet (n=11) and control (n=12) LA-N-1 xenografts used in the first survival experiment, and unpaired Student *t*-test to compare the three xenografts exposed to the longest period of cinacalcet treatment (n=3) to all controls (n=12). Lower panels: Immunohistochemical analysis of GAGE expression conducted on formalin fixed, paraffin-embedded sections of LA-N-1 xenografts exposed to vehicle (VEH) or cinacalcet (CIN). **D.** Network generated with Ingenuity Pathways Analysis to represent pathways modulated in LA-N-1 xenografts upon prolonged treatment with cinacalcet.

First, a genome-wide expression analysis was performed to compare human gene expression profiles in vehicle- and cinacalcet-treated LA-N-1 xenografts. Low stringency analyses (*P*-value≤0.01) identified 61 up-regulated and 32 down-regulated transcripts (Figure 5A and Supplementary Table S2, first sheet). This gene set included only 14 probes with a log fold change value >1 in cinacalcet-treated tumors. Interestingly, 10 (71%) of them hybridized with cancer-testis antigens (CTAs) genes. However, these differences were not statistically significant after *P*-value adjustment. Nonetheless, in this first heat map it was apparent that a group of genes was specifically modulated in the three tumors exposed to the longest period of treatment.

This was confirmed by a second analysis that examined differentially expressed genes in these three specimens exposed to prolonged cinacalcet treatment (C9-C11) compared to the eight control tumors. In this analysis, adjusted *P*-value (false discovery rate, FDR) <0.05 and absolute log fold change >1 were considered significant. This approach identified a gene profile encompassing 34 probes corresponding to 32 up-regulated and 2 down-regulated transcripts (Figure 5B), Supplementary Table S2, second sheet). Among the first group, 11 (32.3 %) probes hybridized with CTAs. These were X-CTAs, i.e. CTAs encoded by genes mapping on chromosome X (*GAGE* family and *SSX4/4B*). The GAGE family was up-regulated in the first analysis and *in vitro* models as well (Table 1). Interestingly, other up-regulated genes in this second heat map included protein kinase C, alpha (*PRKCA*), ryanodine receptor 2 (*RYR2*) and gamma-aminobutyric-A receptor, alpha-3 (*GABRA3*).

Validation of microarrays data and analysis of relevant candidate genes was conducted by RT-qPCR (Table 2, Figure 5C and Supplementary Figure S4A). These analyses included two genes with a level of significance right below the cut-off value in the second heat map given their biological relevance, a non-X CTA, CCCTC-binding factor (zinc finger protein)-like (*CTCFL*), and neurotrophic tyrosine kinase receptor, type 3 (*NTRK3*). Up-regulation of CTAs of the *GAGE* family was further confirmed by immunohistochemistry (Figure 5C).

**Table 2:** Gene expression analyses of neuroblastoma xenografts following cinacalcet treatment conducted by RT-qPCR

Phenotype	Gene	*MYCN* - A			*MYCN* - NA		
		Cinacalcet (n=11) vs vehicle (n=12), *P*	Fold change	Cinacalcet C9-C11 (n=3) vs vehicle (n= 12), *P*	Cinacalcet (n=14) vs vehicle (n= 13), *P*	Fold change	Cinacalcet C13-C14 (n=3) vs vehicle (n=13), *P*
	*CaSR*	ns	-	< 0.0001	ns	-	ns
**Proliferation**	*MYCN*	ns	-	ns	0.012	1.5	0.02
	*ID2*	ns	-	ns	ns	-	ns
	*TERT*	0.045	1.15	ns	ns		ns
**Differentiation**	*NFL*	ns	-	ns	0.0004	1.65	0.007
	*TUBB3*	ns	-	ns	ns	-	ns
	*S100β*	ns	-	ns	ns	-	ns
	*EPHA2*	ns	-	ns	ns	-	ns
	*NTRK1*	ns	-	ns	ns	-	0.012
	*NTRK3*	ns	-	ns	ns	-	ns
	*p75/NTR*	ns	-	ns	ns	-	ns
	*ERBB3*	0.048	1.5	0.046	ns	-	0.009
**ER stress, apoptosis**	*GRP78*	ns	-	0.012	ns	-	ns
	*ATF4*	0.007	1.39	0.023	ns	-	ns
	*CHOP*	ns	-	ns	ns	-	ns
	*TRB3*	0.003	2.02	< 0.0001	ns	-	ns
	*CALR*	0.007	1.45	0.002	ns	-	ns
	*PUMA*	ns	-	ns	ns	-	ns
	*NOXA*	ns	-	ns	ns	-	ns
**EMT/EndoMT/MET**	*CDH1*	ns	-	0.025	ns	-	ns
	*CDH12*	ns	-	ns	ns	-	0.036
	*Snail1*	ns	-	ns	ns	-	ns
	*Slug*	0.006	1.88	ns	ns	-	ns
	*TWIST1*	0.025	1.85	ns	ns	-	0.015
	*TGFβ*	ns	-	0.0005	ns (0.051)	-	ns
	*TGFβ2*	ns	-	0.037	ns	-	0.011
	*TGFβ3*	ns	-	ns	ns	-	ns
	*COL1A1*	0.038	2.07	0.037	ns	-	ns
	*COL3*	ns	-	ns	ns	-	ns
	*VIM*	ns	-	0.006	ns	-	ns
	*CTGF*	ns	-	-	ns	-	ns
	*FN*	ns	-	ns	ns	-	ns
	*KRT19*	ns	-	0.005	ns	-	0.0001
	*MMP2*	0.0004	4.34	0.011	ns	-	ns
	*CDH5*	0.022	4.97	0.014	ns	-	ns
**CTAs**	*CTCFL*	0.038	3.65	0.004	ns	-	ns
	*SSX4/4B*	0.007	1.13	0.0006	ns	-	ns
	*GAGE-P1*	ns	-	0.035	ns	-	ns (0.06)
	*GAGE-P2*	0.025	6.56	0.0005	ns	-	0.0001
	*GAGE-P3*	0.034	9.04	0.012	ns	-	ns
	*MAGE-A2*	0.0003	3.32	< 0.0001	ns (0.051)	-	ns
	*MAGE-A3*	0.002	4.7	0.0007	ns	-	ns
	*NY-ESO-1*	0.049	7.1	0.002	ns	-	ns
**Calcium signaling**	*PRKCA*	ns	-	0.012	ns	-	ns
	*RYR2*	0.018	1.94	0.009	ns	-	0.005
	*ADCY8*	0.021	2.5	0.0004	0.009	2.02	0.0002
**Host microenvironment**	*Angpt1*	ns	*-*	ns			
	*Angpt2*	0.013	2.19	ns	ns	-	0.044
	*Tie1*	0.015	2.69	0.002	ns	-	ns
	*Kdr*	0.013	1.72	ns	ns	-	ns
	*Cdh5*	0.001	2.17	ns			
	α*Sma*	0.018	1.69	ns	ns	-	0.022
	*Pdgfra*	0.015	4.97	ns	ns	-	0.016
	*Cd68*	0.007	1.99	0.003	ns	-	ns
	*Mmp9*	0.003	4.19	ns			
	*Tgfb*	0.006	2.48	ns	ns	-	ns

*MYCN*-A: *MYCN*amplified

*MYCN*-NA: *MYCN* non-amplified

EMT/EndoMT: Epithelial/endothelial-to-mesenchymal transition.

MET: Mesenchymal-to-epithelial transition.

ns: not significant

CTAs: Cancer-testis antigens

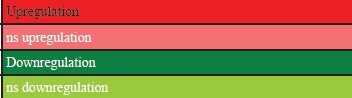

Gene expression analyses were performed by RT-qPCR. Two-tailed Mann-Whitney *U* test was used to compare all cinacalcet and control specimens in each experiment. Unpaired Student *t*-test was performed to compare the three xenografts exposed to the longest period of cinacalcet treatment (n=3) to all controls (n=12).

As shown in Table 2 and Supplementary Figure S4A, besides *SSX4/4B* and the *GAGE* family, other X-CTAs (*MAGE-A2*, *MAGE-A3* and *NY-ESO-1*) and *CTCFL,* were up-regulated in *MYCN*-amplified specimens exposed to prolonged cinacalcet treatment. In PDX, only CTAs of the *GAGE* family were up-regulated. Increased expression of genes involved in differentiation, ER stress and EMT was also found in both models, but more robustly in *MYCN*-amplified xenografts.

To identify relevant biological processes promoted by cinacalcet in neuroblastoma, Gene Ontology (GO) enrichment analysis was conducted (Supplementary Table S3, first sheet). Overrepresented processes among up-regulated genes were calcium-signaling pathways and ion transport (adenylyl cyclase type 8 -*ADCY8-*, *RYR2* and *PRKCA*), cell adhesion, differentiation (*GABRA3*, *NTRK3*) and also CTAs. Among down-regulated genes, which were less abundant, transcription factors were overrepresented, notably inhibitor of differentiation 2 -*ID2*- and v-myb avian myeloblastosis viral oncogene homolog -*MYB*-.

Next, a search for molecular pathways in which those genes might participate was conducted with Ingenuity Pathways Analysis (IPA). The most significant network included up-regulation of *RYR2* and *NTRK3,* together with down-regulation of *ID2* and fibronectin *-FN1-* (Figure 5D and Supplementary Table S3, second sheet). Other top networks were involved in gene regulation, cell death and survival.

Genome-wide expression analyses of murine genes did not find statistically significant differences between vehicle- and cinacalcet-treated xenografts (Supplementary Table S2, third sheet, and Supplementary Figure S5A). However, Gene Ontology (Supplementary Table S3, third sheet) and IPA (Supplementary Figure S5B and Supplementary Table S3, fourth sheet) analyses showed gene expression changes consistent with microenvironment remodeling and angiogenesis, most notably *jagged-1*, *semaphorin-5B* and *neuropilin-2*.

Consequently, a number of candidate genes were analyzed by RT-qPCR taking into account that both human and murine cells seemed to participate in microenvironment remodeling. Down-regulation of human vascular specific cadherin *CDH5* and up-regulation of mesenchymal markers (*COL1A1*, *VIM*) was detected following cinacalcet treatment. More prominent changes of murine gene expression consistent with angiogenesis and fibrosis were seen in cinacalcet-treated *MYCN*-amplified xenografts (Table 2).

Finally, RT-qPCR detected a moderate up-regulation of *CaSR* mRNA upon prolonged treatment with cinacalcet in *MYCN*-amplified xenografts (Table 2, Supplementary Figure S4B). This was confirmed at the protein level by Western blot (Supplementary Figure S4C). In PDX, only a modest up-regulation was noted at the mRNA level (Table 2).

Altogether, *in vivo* data were consistent with those produced *in vitro* and supported that prolonged exposure to cinacalcet induces ER stress, differentiation and fibrosis in neuroblastoma together with upregulation of CTAs.

## DISCUSSION

Refractory neuroblastomas remain a remarkable clinical challenge and novel therapeutic strategies are needed to change the uniformly fatal outcome of these patients. Our present data show a targeted therapy that reduces neuroblastoma tumor growth and induces up-regulation of CTAs, thus paving the way for novel immunotherapeutic approaches and combination treatments.

Our first analyses were based on our previous findings and showed that acute exposure to cinacalcet triggers ER stress and apoptosis in CaSR-positive, *MYCN*-amplified neuroblastoma cells, dependent on phospholipase C activation. Massive calcium release from the ER is a potent inducer of ER stress, and cancer cells are especially susceptible to this injury [22]. We focused on GRP78-ATF4-PeIF2α as this pathway has been reported to mediate ER stress-induced cell death in neuroblastoma [20]. Induction of these molecules was followed by up-regulation of *CHOP* and *TRB3*, an indication of ER stress coupled to cell death [23]. Also, according to an ER-centered mechanism of apoptosis, cinacalcet activated caspase-4, together with other caspases of the intrinsic apoptotic pathway [19]. In keeping with literature, *MYCN*-non amplified cells were less sensitive to ER stress [20].

Microarrays analyses provided *in vivo* evidence that cinacalcet triggers ER stress in neuroblastoma, including up-regulation of *RYR2*. Intracellular Ca^2+^ leak via RYR2 and consequent depleted ER stores have been associated with ER stress in pancreatic β cells [24]. Up-regulation of *ADCY8* represented another *in vivo* indication of Ca^2+^ leak from the ER. In non-excitable cells, ADCY8 is predominantly stimulated by Ca^2+^ ions entering the cells via capacitative calcium entry, a mechanism triggered by depletion of intracellular Ca^2+^ stores [25]. Moreover, *CNBD1* (cyclic nucleotide-binding domain-containing protein 1) was also up-regulated in the second heat map [26]. Further studies will be necessary to establish whether increased expression of this domain is an indication of cAMP production by ADCY8 or whether CaSR might couple to G_s_ in this cellular context.

We next evaluated the effects of sustained *in vitro* exposure to cinacalcet. Apoptosis was also detected in *MYCN*-amplified cells. Other mechanisms of cell death, including necroptosis, might concur. Moreover, surviving CaSR-positive cells, irrespective of *MYCN* status, displayed morphological signs and expression patterns consistent with differentiation induction and cell cycle exit. These effects were more pronounced in cells with a less differentiated phenotype [21]. Microarrays data also showed up-regulation of two genes associated with benign neuroblastomas, *GABRA3* [27] and *NTRK3* [28], following prolonged exposure to cinacalcet. Concurrently, *ID2, MYB* and *FN1* were down-regulated. ID2 is a helix-loop-helix transcription factor that acts under the control of MYC proteins, blocks differentiation and stimulates cell proliferation [29]. MYB is a transcription factor that cooperates with MYCN in cell cycle regulation of *MYCN*-amplified neuroblastomas [30]. By RT-qPCR, we also found a moderate down-regulation of *MYCN* in PDX and *in vitro* models. Down-regulation of *MYCN*, *ID2, TERT* and *FN1* has been also reported in neuroblastoma cells undergoing differentiation upon exposure to retinoids [7-10]. Induction of *TERT* in LA-N-1 xenografts might be associated with fibrosis [31]. Interestingly, up-regulation of synaptopodin-2 (*SYNPO*) could be an additional indication of *MYCN* down-regulation, as its main upstream regulator, DKK3, is negatively modulated by MYCN [32, 33].

Prolonged *in vitro* exposure to cinacalcet reproduced quite notably other effects induced by this drug *in vivo*, namely up-regulation of CTAs. This good correlation between our *in vitro* and *in vivo* findings makes less likely that hypocalcemia and associated hormonal changes account for the phenotypes observed *in vivo*. CTAs encompass a large family of antigens that are considered ideal targets for immunotherapy given their almost tumor-specific pattern of expression and strong immunoreactivity [34]. However, the efficacy of CTA-based immunotherapies is hampered by the remarkable inter- and intra-tumoral heterogeneity expression of these antigens. Attempts to increase their expression have relied on epigenetic modifiers, taking into account the primary mechanism regulating CTAs transcription [35]. To our surprise, cinacalcet induced a robust up-regulation of several CTAs in all neuroblastoma models examined. Up-regulated CTAs were mainly encoded by genes mapping on chromosome X (*GAGE*, *MAGE*, *SSX4* families and *NY-ESO-1*), which are the most immunogenic, and less significantly a non-X CTA, *CTCFL*. It has been reported that p53 strongly represses *CTCFL* expression [36]. Accordingly, *CTCFL* was only up-regulated in the *TP53*-null model. Given that *CTCFL* regulates other CTAs, mainly the *MAGE* family [37], it is also likely that lack of *MAGEs* up-regulation in cinacalcet-treated PDX is partially due to the normal activity of p53 in these tumors.

Expression of *GAGE* antigens has been reported in neuroblastoma [38]. However, the roles of CTAs in cancer remain ill-defined, and some evidences indicate that they support cancer cell survival, invasion or migration [39], but expression of CTAs has been associated with better outcome in glioblastoma patients [40]. Our results show up-regulation of CTAs in xenografts displaying slower growth rates and in three *in vitro* models undergoing differentiation upon cinacalcet exposure. Thus, while further studies are warranted to establish the function of these molecules in cancer, up-regulation of CTAs in our models is not associated with increased neuroblastoma aggressiveness. Moreover, this effect might promote higher effectiveness of CTA-based immunotherapies and provide surrogate circulating markers of neuroblastoma response to cinacalcet.

CaSR activation has been described to promote cardiac fibrosis [41]. In contrast, cinacalcet seems to reduce collagen deposition in models of kidney failure [42] and down-regulation of mesenchymal markers is also associated with CaSR activation in colon cancer cell lines [43]. In *MYCN*-amplified xenografts, cinacalcet treatment was associated with induction of fibrosis while normal organs were not affected. Indeed, fibrosis of refractory tumors would be clinically beneficial. Apoptosis and ER stress could be among initiating events. Although apoptosis was originally described to eliminate cells without disturbing surrounding tissues, more recent evidences support that it might promote fibrosis [44]. Chronic ER stress is also a potent pro-fibrotic stimulus [45]. Although host cells were noticeably cooperating in microenvironment remodeling, a number of transcripts associated with EMT or mesenchymal-to-epithelial transition were modulated *in vitro* as well. The complex gene expression patterns observed most likely reflect overlapping phases of fibrosis taking place simultaneously given the persistent, but intermittent, nature of the triggering injury. Nevertheless, the final output indicates that cinacalcet is mostly a pro-fibrotic stimulus in *MYCN*-amplified neuroblastomas. A factor potentially contributing to the lesser extent of fibrosis in PDX, besides being less susceptible to ER stress, is normal p53 function [46].

Last but not least, cinacalcet also promoted CaSR up-regulation. This finding is in accordance with literature [47] and would be clinically relevant should cinacalcet be used as a treatment for this tumor. Most unfavorable neuroblastomas exhibit low levels of CaSR expression, and enforced expression of the receptor notably reduces neuroblastoma cell proliferation and tumorigenicity [15]. Thus, increase of CaSR expression upon cinacalcet treatment would reduce neuroblastoma aggressiveness by itself, and additionally promote a more pronounced response to the drug over time.

In summary, we show that cinacalcet inhibits neuroblastoma tumor growth by promoting differentiation, ER stress, apoptosis and/or fibrosis depending on time of exposure and cellular context. Cinacalcet also up-regulates CTAs in neuroblastoma thus providing novel opportunities for CTA-based immunotherapies and circulating surrogate markers of neuroblastoma response to this treatment.

## MATERIALS AND METHODS

### Cell lines

Seven neuroblastoma cell lines (LA-N-1, LA1-55n, SH-SY5Y, SK-N-JD, SK-N-LP, LA1-5s and SK-N-AS) exhibiting different degrees of differentiation [21], HEK-293 cells and human fibroblasts were obtained from the repository at Institut de Recerca Pediàtrica - Hospital Sant Joan de Déu (Barcelona, Spain). Unless otherwise specified, they were grown in Roswell Park Memorial Institute (RPMI)-1640 medium supplemented with 10% fetal bovine serum (Invitrogen, Carlsbad, CA), 2 mM L-glutamine, penicillin (100 U/mL) and streptomycin (100 μg/mL), at 37°C and 5% CO_2._. Mycoplasma polymerase chain reaction (PCR) tests were routinely performed. Characterization of cell lines included analysis of *MYCN* amplification [14], *TP53* sequence and authentication by STR profiles.

### RNA isolation, cDNA synthesis, PCR and qPCR

Total RNA was isolated using TriReagent (Sigma, St Louis, MO). Retrotranscription, PCR and qPCR were carried out as described [14, 15]. Real-time PCR runs were performed in a 7500 SDS system using gene-specific Assays on Demand and Taqman Universal PCR Master Mix, or specific primers (Supplementary Table S1) and SYBRGreen (Applied Biosystems, Forster City, CA). Relative expression levels were calculated according to the 2^−ΔΔCt^ method using *TATA-box binding protein* (*TBP*) as normalizing gene. Only samples with a *TBP* Ct lower than 30 were used to ensure RNA and cDNA quality. Quantitative PCR runs conducted according to these procedures were shown to produce highly consistent data (coefficient of variation < 5%, not shown).

### Cell viability and proliferation assays

Cells were plated into 96-well plates in RPMI-1640 10% FBS. Six replicate wells were seeded for each cell line and condition. Next day, they were treated with cinacalcet (Selleckchem, Houston, TX), NPS R-568 (Tocris, Minneapolis, MN) or DMSO. Viability was measured with CellTiter^96^ Aqueous Cell Proliferation Assay (Promega, Madison, WI) 72 hours later. IC_50_ (i.e. drug concentrations achieving 50% decrease in cell viability) were determined with GraphPad Prism software. The same experiments were performed in the presence or absence of 20 μM Z-VAD-FMK (Promega). Proliferation rates were assessed as reported [15].

To examine the combined effects of cinacalcet and U73122 (Sigma) on cell viability, cells were plated in 24-well plates in RPMI-1640 10% FBS. Four replicate wells were seeded for each cell line and condition. Next day, media were replaced by calcium-free Dulbecco's modified Eagle's medium (DMEM) (Invitrogen) supplemented with bovine serum albumin (0.2% w/v), 4 mM L-glutamine and 0.5 mM CaCl_2_ (these conditions will be mentioned as serum deprivation media) for 16 hours. Cultures were then exposed to cinacalcet in the same media containing 0.5 mM or 3 mM CaCl_2_ and/or U73122. Cell viability was determined as above.

### Apoptosis assays

Cells were seeded in RPMI-1640 10% FBS. Next day, they were grown in serum deprivation media for 16 hours. Cultures were then exposed to cinacalcet for 8 or 24 hours in the same media containing 0.5 mM or 3 mM CaCl_2_. Floating and adherent cells were collected and stained with Annexin V-FITC Detection Kit (Life Technologies, Carslbad, CA) as per manufacturer's instructions. Cells were analyzed by flow cytometry on a FACSCalibur System and results were processed with CellQuest software (Becton Dickinson, Mountain View, CA).

### Immunoblots

Cells were exposed to cinacalcet or DMSO, collected in ice-cold PBS and lysed in 10 mM Tris-HCl pH=6.8, 1 mM EDTA, 150 mM NaCl, 1% SDS. Thirty to fifty μg proteins were electrophoresed in 8-14% SDS-PAGE and transferred onto nitrocellulose membranes. Incubation with primary antibodies (caspase-3, caspase-4, caspase-7, caspase-9, cleaved PARP, eIF2α, P-eIF2α, GRP78 (Cell Signaling Technologies, Danvers, MA), CaSR (Abcam, Cambridge, UK), ATF4 (Santa Cruz Biotechnology, Dallas, TX), α-tubulin (Sigma)) was followed by horseradish peroxidase-conjugated secondary antibodies (Promega). Immunoreactive bands were detected with enhanced chemiluminiscence reagents (Amersham Pharmacia, Piscataway, NJ). Alternatively, secondary antibody IRDye680RD goat-anti-mouse IgG (Li-COR #926-68070) or IRDye800CW goat-anti-rabbit IgG (Li-COR #926-32211) were used and blots were visualized with Li-COR Odyssey system (Li-COR Biosciences, Lincoln, NE).

### Mouse xenograft models

Procedures were approved by the Institutional Animal Research Ethics Committee. Two *in vivo* models were generated in four to six-week-old female athymic Nude-Foxn1 *nu*/*nu* mice (Charles River, Wilmington, MA). LA-N-1 cells generated a CaSR-positive model bearing *MYCN* amplification and mutation of *TP53*. A tumor fragment was obtained from the liver metastasis of a stage 4 neuroblastoma without these two genetic alterations. Written informed consent was obtained and procedures were approved by the Institutional Review Board. The tumor was subcutaneously inoculated and allowed to grow up to 2 cm^3^ to generate a patient-derived xenograft (PDX).

LA-N-1 cells (10^7^) resuspended in Matrigel:PBS (Becton Dickinson) or PDX fragments were subcutaneously inoculated. Tumors were allowed to grow until dimensions reached 7×7 mm. Mice were then randomized to receive either vehicle or cinacalcet (10 mg/kg/day) by oral gavage 6 days per week until tumor volume reached 2 cm^3^. Cinacalcet was prepared with Mimpara® tablets. Following homogenization in a mortar, the resulting powder was reconstituted with 0.5% Tween 20 (20%, v/v) and carboxymethylcellulose (0.25%, w/v) to a final concentration of 20 mg/mL. Each dose contained 5 μL of this mixture per gram of mouse weight. Dimensions of tumors were measured thrice a week using a digital caliper. Tumor volume was calculated as L × W^2^/2 in which “L” indicates length in mm and “W” indicates width in mm. At the end of the experiment, tumors were excised and half of each specimen was frozen in liquid nitrogen and the other was fixed in 10% formalin.

### Ionized calcium concentrations in blood

Blood samples (100 μL) were collected from facial veins of control and cinacalcet-treated mice once a week during the first six weeks of treatment. Extractions were performed before drug/vehicle administration. Ionized calcium concentrations were measured using an EPOC Reader (Alere Healthcare, Waltham, MA).

### Immunohistochemistry

Sections (4 μm) of formalin fixed, paraffin-embedded tumors were stained with haematoxylin-eosin (H&E), Masson's trichrome or processed for immunohistochemistry to analyze CaSR expression as described [14]. A similar protocol was carried out with anti-GAGE7 (Life Technologies) and anti-human nuclei, clone -3E1.3 (Merck-Millipore, Darmstadt, Germany).

### Microarray processing and analysis

Total RNA was isolated from 8 xenografts exposed to cinacalcet and 8 receiving vehicle in the first survival experiment. These were the last 8 tumors excised in each group. Genome-wide expression analyses were performed using Affymetrix Human Gene Array 2.1 ST and Mouse Gene Array 2.1 ST (Affymetrix, Santa Clara, CA) as per manufacturer's protocol. Microarrays data were analyzed at the Statistics and Bioinformatics Unit of Vall d'Hebron Research Institute (Barcelona, Spain). After standard quality controls, a cinacalcet-treated sample (C6) was considered an outlier under the criteria of distance between arrays and intensity distribution [48] and excluded from further analyses. Robust Multi-array Average (RMA) algorithm was used for pre-processing microarray. In order to minimize the effect of *P*-value adjustment that multiple testing implies, normalized data were subjected to non-specific filtering to remove low (50%) signal and low (50%) variability genes. Selection of differentially expressed genes was based on a linear model analysis with empirical Bayes modification for the variance estimates. To account for multiple testing, *P*-values were adjusted to obtain stronger control over the false discovery rate (FDR). Differentially expressed genes (*P*-value ≤0.01) obtained from comparing the three xenografts exposed to the longest treatment with cinacalcet (C9-C11) to all control tumors were used as input for a gene enrichment analysis against the Gene Ontology [49] using DAVID tool, which performs Fisher Exact tests to compare the proportion of significant genes found on each GO category [50]. A more restrictive gene set (adjusted *P*-value ≤0.25 and absolute log fold change >0.5) was analyzed using QIAGEN's Ingenuity® Pathway Analysis. Microarray data have been deposited in the Gene Expression Omnibus database (Accession number GSE73509).

### Statistical analysis

Other statistical analyses were performed with SPSS v20 and GraphPad Prism 5 softwares. Comparison of means was performed by Student *t* or Mann-Whitney *U* tests. For event-free survival (EFS) analysis, an event was defined as tumor size that exceeded 2 cm^3^. The log-rank statistic was used to compare EFS probabilities between groups. *P*<0.05 was considered significant.

## SUPPLEMENTARY FIGURES AND TABLES








