# Supplementary material for: Cinacalcet inhibits neuroblastoma tumor growth and upregulates cancer-testis antigens
Source: Oncotarget. 2016 Feb 17;7(13):16112–29. doi: 10.18632/oncotarget.7448 (PMC4941301; doi:10.18632/oncotarget.7448)
Supplement: Supplementary file 1 [file oncotarget-07-16112-s001.pdf]

SUPPLEMENTARY FIGURES AND TABLES

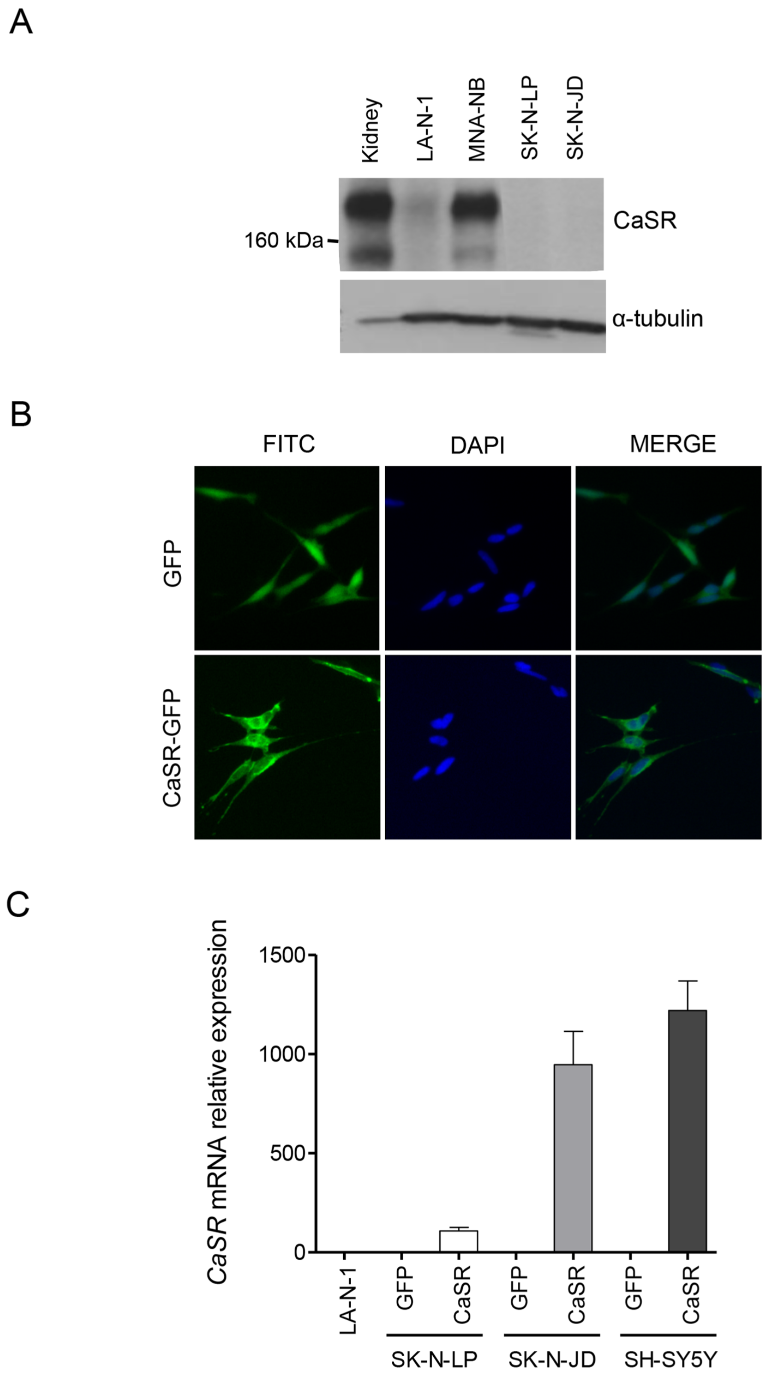

Supplementary Figure S1: CaSR expression in neuroblastoma models. A. Proteins were isolated from three neuroblastoma cell lines and from the liver metastasis of a *MYCN*-non amplified neuroblastoma (MNA-NB) that generated a patient-derived xenograft. Western blot was conducted to analyze CaSR expression. Kidney was used as positive control. **B.** Fluorescence microphotography showing SH-SY5Y cells following stable transfection with pCMV-GFP or pCMV-CaSR-GFP. **C.** Total RNA was isolated from LA-N-1 cells, and SK-N-LP, SK-N-JD and SH-SY5Y cell lines following stable transfection with pCMV-GFP or pCMV-CaSR-GFP. Analysis of *CaSR* mRNA expression was conducted by RT-qPCR. Expression levels were calculated as a fold change relative to mRNA expression detected in LA-N-1 cells and are the mean of two independent qPCR runs.

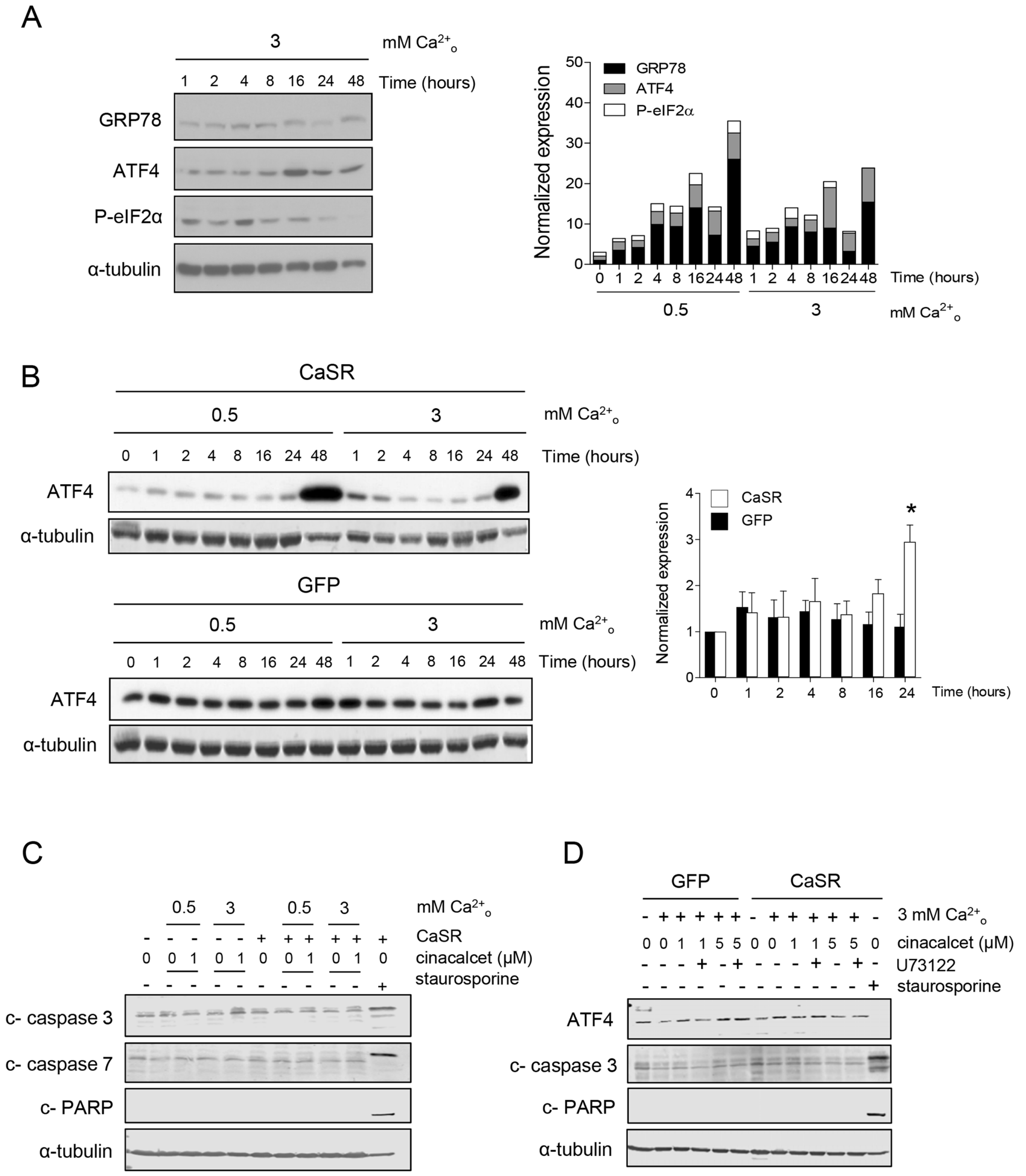

Supplementary Figure S2: Cinacalcet induces ER stress in CaSR-positive, *MYCN*-amplified neuroblastoma cell lines. **A.** SK-N-LP cells stably transfected with pCMV-CaSR-GFP were exposed to 6 µM cinacalcet following serum deprivation in 3 mM $CaCl_2$ for indicated periods of time and lysed for Western blot analyses. Densitometry was carried out to quantify protein expression relative to α-tubulin levels (right). **B.** SK-N-LP cells stably transfected with pCMV-GFP or pCMV-CaSR-GFP were exposed to 1 µM cinacalcet for indicated periods of time as described in panel A, and lysed for Western blot analysis. Graph (right) shows mean values of three independent experiments. ATF4 increase at 24 hours was statistically significant ($P < 0.01$, two-tailed Student's *t*-test). **C.** SH-SY5Y cells stably transfected with pCMV-GFP or pCMV-CaSR-GFP were exposed to cinacalcet in serum deprivation media or 1 µM staurosporine for 24 hours in the presence of 0.5 or 3 mM $CaCl_2$. Cells were lysed to perform immunoblots. **D.** CaSR-positive and -negative SH-SY5Y cells were exposed to cinacalcet after serum deprivation as described in C, in the presence or absence of 100 nM U73122 and then lysed to conduct Western blots. Data showed in panels A, C and D are representative of at least two independent experiments.

A

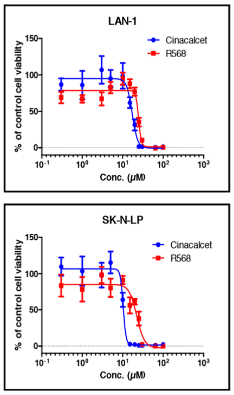

	IC50 (μM)		
	Cinacalcet	NPS R-568	*P*
LA-N-1	17.19	24.81	< 0.0001
SK-N-LP	10.31	22.38	< 0.0001
SK-N-JD	10.92	25.82	< 0.0001
LA1-5s	16.73	34.5	< 0.0001
LA1-55n	10.52	11.42	Not significant
SK-N-AS	10.26	15.14	Not significant
Fibroblasts	15.52	nd	
HEK-293	14.73	nd	

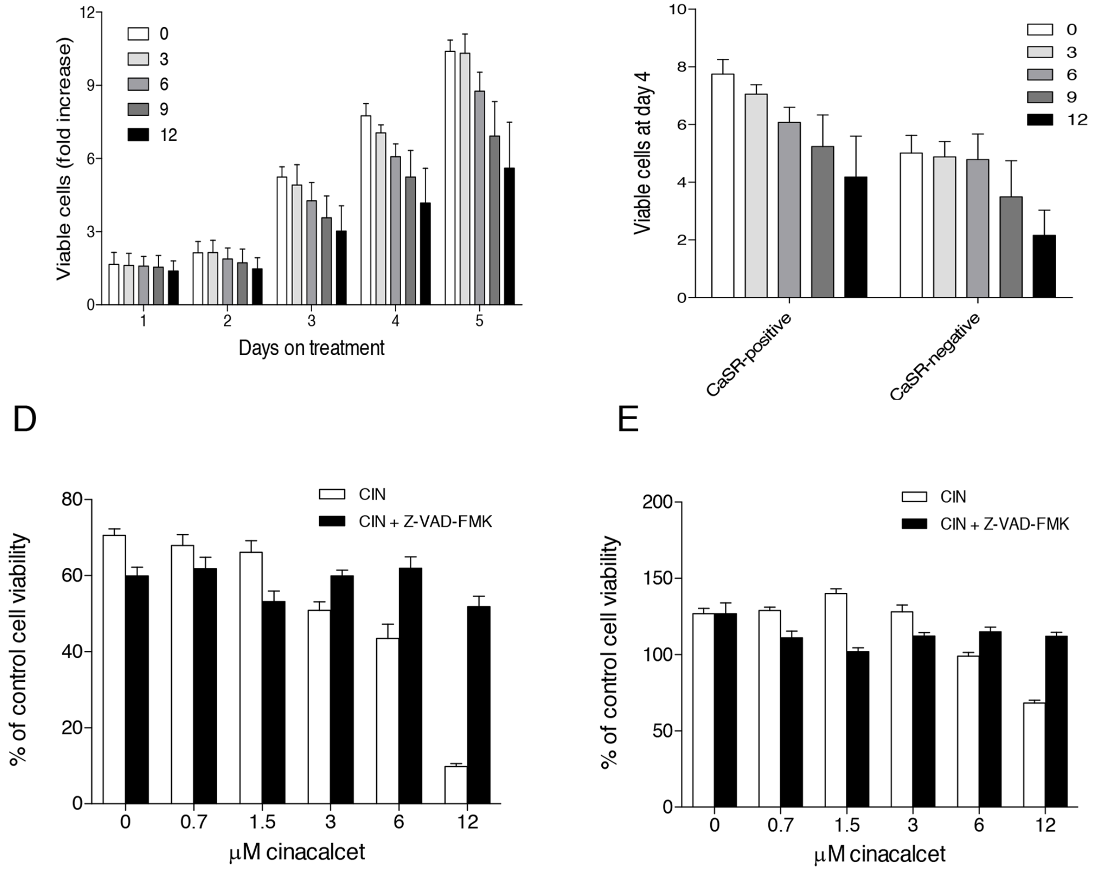

Supplementary Figure S3: Neuroblastoma cell viability following exposure to cinacalcet. A. Six neuroblastoma cell lines, HEK-293 cells and human fibroblasts were exposed cinacalcet or another calcimimetic, NPS R-568, in 96-well plates for 72 hours. Six replicate wells were seeded for each cell line and condition. Cell viability was measured with CellTiter[96] Aqueous Cell proliferation Assay. IC_{50} concentrations were determined with GraphPad Prism software. **B.** LA-N-1 cells were seeded in 96-well plates and exposed to cinacalcet (doses ranging from 3 to 12 μM) for 5 days. Cells were stained daily with crystal violet and proliferation rates were calculated as a fold increase of absorbance measurement relative to day 1. **C.** The effect of cinacalcet on proliferation rates, measured as in panel B, was compared in CaSR-positive cells (LA-N-1 and SK-N-JD CaSR-positive cells) and CaSR-negative (SK-N-JD and SK-N-AS) cells. The mean of two experiments is shown. **D.** LA-N-1 cells were exposed to cinacalcet in the presence or absence of 20 μM Z-VAD-FMK for 48 hours. Cell viability was measured with MTS assays. **E.** CaSR-negative SK-N-JD cells were grown and analyzed in the conditions described in panel D. Data presented in the five panels are representative of at least two independent experiments.

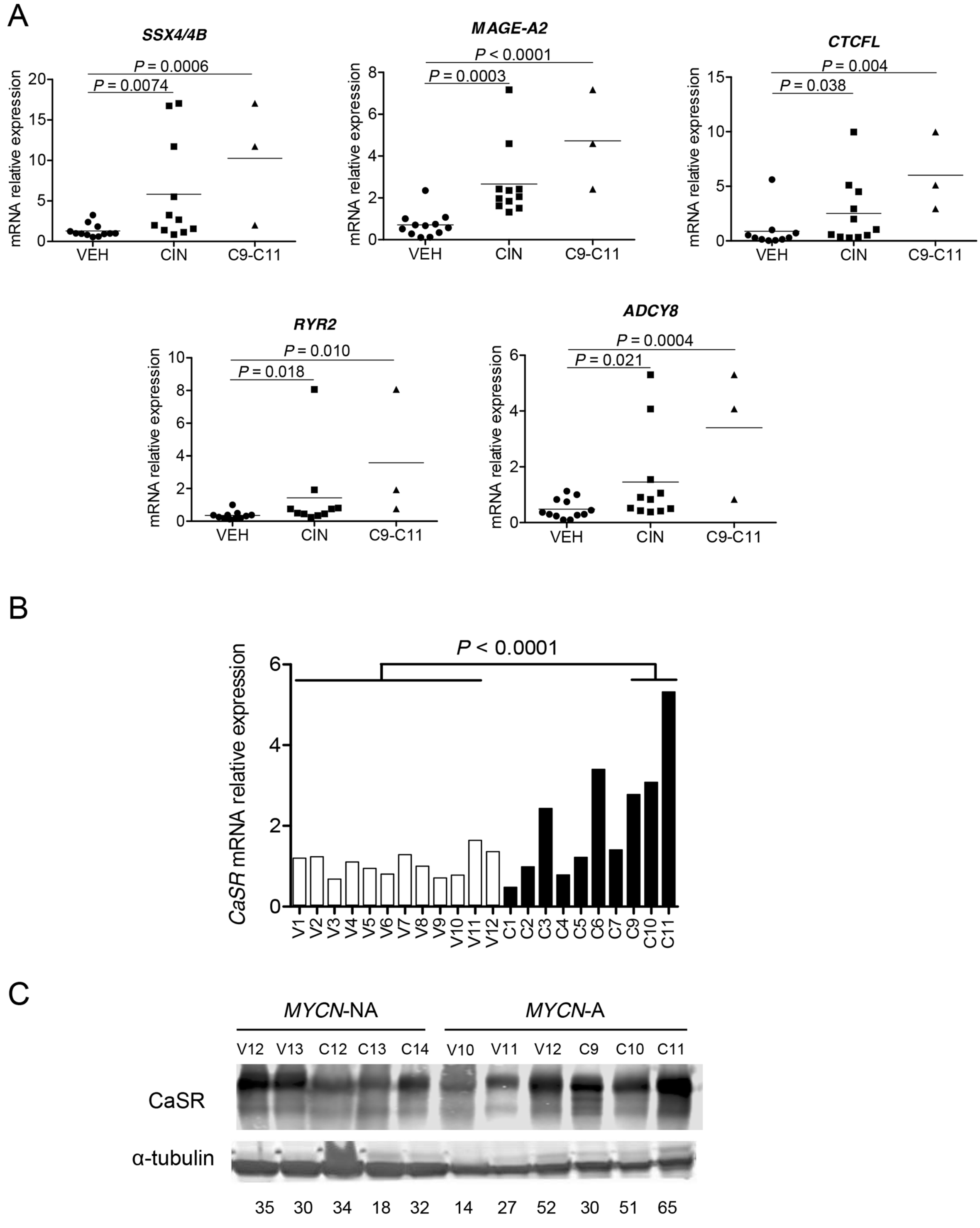

Supplementary Figure S4: Gene expression analyses in cinacalcet-treated neuroblastoma xenografts. A. Total RNA was isolated from LA-N-1 xenografts of the first survival experiment. Gene expression analyses were performed by RT-qPCR. Statistical significance was calculated using two-tailed Mann-Whitney *U* test to compare all cinacalcet- (n=12) and vehicle-treated (n=11) xenografts. Unpaired Student *t*-test was used to compare the three xenografts exposed to the longest period of cinacalcet treatment (C9-C11) to all controls (n=12). **B.** *CaSR* mRNA was analyzed by RT-qPCR in vehicle- and cinacalcet-treated LA-N-1 xenografts. **C.** Western blot was conducted to analyze CaSR protein expression in *MYCN*-amplified xenografts and PDX.

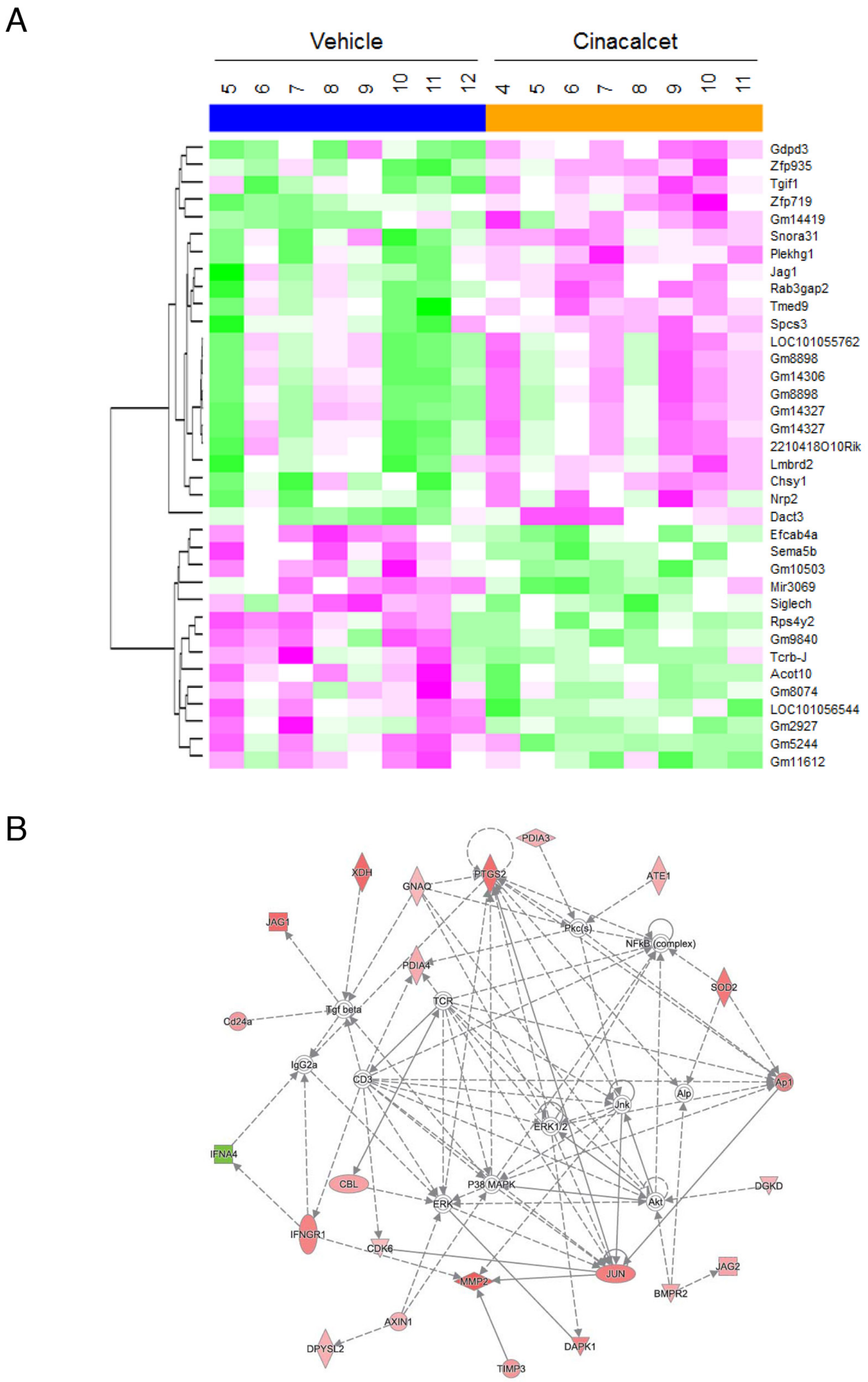

Supplementary Figure S5: Genome-wide expression analyses of murine genes in cinacalcet-treated LA-N-1 xenografts. **A.** Total RNA was isolated from LA-N-1 xenografts that received either cinacalcet or vehicle in the first survival experiment. Heat map depicts murine gene expression patterns in these samples. **B.** Network generated with Ingenuity Pathways Analysis to represent modulated pathways in murine cells upon cinacalcet treatment.

Supplementary Table S1: Primers sequences and assays-on-demand used for RT-qPCR.

See Supplementary File 1

Supplementary Table S2: First sheet: Genome-wide human gene expression analysis in LA-N-1 xenografts exposed to vehicle (n=8) or cinacalcet (n=7). **Second sheet:** Genome-wide human gene expression analysis in LA-N-1 xenografts exposed to vehicle (n=8) or prolonged treatment with cinacalcet (n=3, C9-C11). **Third sheet:** Genome-wide murine gene expression analysis in LA-N-1 xenografts exposed to vehicle (n=8) or cinacalcet (n=7).

See Supplementary File 2

Supplementary Table S3: First sheet: Gene Ontology (GO) analysis to identify biological processes in modulated genes upon prolonged exposure to cinacalcet in LA-N-1 xenografts. **Second sheet:** Ingenuity Pathways Analysis to identify molecular pathways promoted in LA-N-1 xenografts upon prolonged exposure to cinacalcet. **Third sheet:** GO analysis to identify biological processes in murine genes modulated in LA-N-1 xenografts following cinacalcet treatment. **Fourth sheet:** Ingenuity Pathways Analysis to identify molecular pathways in murine gene expression changes promoted by cinacalcet in LA-N-1 xenografts.

See Supplementary File 3
